# Supplementary material for: Serum Anti-Aging Protein α-Klotho Mediates the Association between Diet Quality and Kidney Function
Source: Nutrients. 2023 Jun 14;15(12):2744. doi: 10.3390/nu15122744 (PMC10301566; doi:10.3390/nu15122744)
Supplement: Supplementary file 1 [file nutrients-15-02744-s001.zip › nutrients-2438534-supplementary.pdf]

Table S1. Criteria for scoring each component of Healthy Eating Index-2015.

HEI-2015 Components	Points Range	Scoring Standard [a]	
		**Maximum**	**Minimum**
Adequacy Components			
Total Fruits [b]	0–5	≥0.8 cup equiv. per 1000 kcal	0
Whole Fruits [c]	0–5	≥0.4 cup equiv. per 1000 kcal	0
Total Vegetables [d]	0–5	≥1.1 cup equiv. per 1000 kcal	0
Greens and Beans [d]	0–5	≥0.2 cup equiv. per 1000 kcal	0
Whole Grains	0–10	≥1.5 oz equiv. per 1000 kcal	0
Dairy [e]	0–10	≥1.3 cup equiv. per 1000 kcal	0
Total Protein Foods [f]	0–5	≥2.5 oz equiv. per 1000 kcal	0
Seafood and Plant Proteins [e, g]	0–5	≥0.8 oz equiv. per 1000 kcal	0
Fatty Acids [h]	0–10	(PUFAs + MUFAs)/SFAs ≥2.5	0
HEI-2015 Moderation			
Refined Grains	10–0	≤1.8 oz equiv. per 1000 kcal	≥4.3 oz equiv. per 1000 kcal
Sodium	10–0	≤1.1 grams per 1000 kcal	≥2.0 grams per 1000 kcal
Added Sugars	10–0	≤6.5% of energy	≥26% of energy
Saturated Fats	10–0	≤8% of energy	≥16% of energy

[a] Intakes between the minimum and maximum standards are scored proportionately. [b] Includes 100% fruit juice.
[c] Includes all forms except juice.
[d] Includes legumes (beans and peas)
[e] Includes all milk products, such as fluid milk, yogurt, and cheese, and fortified soy beverages.
[f] Includes legumes (beans and peas)
[g] Includes seafood, nuts, seeds, soy products (other than beverages), and legumes (beans and peas).
[h] Ratios of poly-and monosaturated fatty acids (PUFAs and MUFAs) to saturated fatty acids (SFAs).
Abbreviation: MUFA, monounsaturated fatty acids; PUMA, polyunsaturated fatty acids.

Table S2. Association between standardized HEI-2015 scores and log-transformed UACR among 12,817 participants aged 40-79 years in the NHANES.

	Weighted β coefficient (95%CI) for log-transformed UACR		
	**Model 1**	**Model 2**	**Model 3**
Overall HEI-2015	-0.10 (-0.12, -0.08) *	-0.06 (-0.08, -0.04) *	-0.05 (-0.08, -0.04) *
Total fruits	-0.07 (-0.09, -0.05) *	-0.04 (-0.06, -0.02) *	-0.03 (-0.05, -0.01) *
Whole fruits	-0.07 (-0.10, -0.05) *	-0.05 (-0.07, -0.02) *	-0.04 (-0.06, -0.02) *
Total vegetables	-0.04 (-0.06, -0.02) *	-0.02 (-0.04,0.01)	-0.02 (-0.04, -0.01) *
Greens and beans	-0.08 (-0.10, -0.06) *	-0.06 (-0.08, -0.04) *	-0.05 (-0.07, -0.03) *
Whole grain	-0.05 (-0.07, -0.03) *	-0.04 (-0.06, -0.02) *	-0.04 (-0.06, -0.02) *
Refined grain	-0.06 (-0.08, -0.04) *	-0.05 (-0.07, -0.03)	-0.02 (-0.04, -0.01) *
Total dairy	-0.02 (-0.04, 0.01)	-0.01 (-0.03, 0.01)	-0.01 (-0.03, 0.01)
Total protein	-0.01 (-0.03, 0.01)	0.01 (-0.02, 0.02)	-0.01 (-0.03, 0.01)
Seafood and plant protein	-0.07 (-0.09, -0.05) *	-0.04 (-0.06, -0.02) *	-0.04 (-0.06, -0.02) *
Fatty acid	-0.03 (-0.05, -0.01) *	-0.01 (-0.03, 0.01)	-0.01 (-0.03, 0.01)
Saturated fat	-0.02 (-0.04, -0.01) *	-0.03 (-0.05, -0.01) *	-0.01 (-0.03, 0.01)
Sodium	-0.05 (-0.07, -0.03) *	-0.05 (-0.07, -0.03) *	-0.01 (-0.03, 0.01)
Add sugar	0.04 (0.02, 0.06) *	0.06 (0.04, 0.08) *	0.03 (0.01, 0.05) *

Model 1. Adjusted for age, sex and race
Model 2. Model plus education, income, total energy intake, alcohol intake, smoking, and physical activity
Model 3. Model 2 plus BMI, self-reported hypertension, self-reported diabetes, and self-reported cardiovascular disease
*p<0.05

Table S3. Mediating role of α-klotho in the association between standardized HEI-2015 scores and log-transformed UACR among 12,817 participants aged 40-79 years in the NHANES.

	Weighted β coefficient (95%CI) for log-transformed UACR		
**Mediator: α-Klotho**	**Total effect**	**Direct effect**	**Indirect effect**
Overall HEI-2015	-0.05 (-0.08, -0.04) *	-0.05 (-0.08, -0.04) *	-
Total fruits	-0.03 (-0.05, -0.01) *	-0.03 (-0.05, -0.01) *	-
Whole fruits	-0.04 (-0.06, -0.02) *	-0.04 (-0.06, -0.02) *	-
Total vegetables	-0.02 (-0.04, -0.01) *	-0.02 (-0.04, -0.01) *	-
Greens and beans	-0.05 (-0.07, -0.03) *	-0.05 (-0.07, -0.03) *	-
Whole grain	-0.04 (-0.06, -0.02) *	-0.04 (-0.06, -0.02) *	-
Refined grain	-0.02 (-0.04, -0.01) *	-0.02 (-0.04, -0.01) *	-
Total dairy	-0.01 (-0.03, 0.01)	-0.01 (-0.03, 0.01)	-
Total protein	-0.01 (-0.03, 0.01)	-0.01 (-0.03, 0.01)	-
Seafood and plant protein	-0.04 (-0.06, -0.02) *	-0.04 (-0.06, -0.02) *	-
Fatty acid	-0.01 (-0.03, 0.01)	-0.01 (-0.03, 0.01)	-
Saturated fat	-0.01 (-0.03, 0.01)	-0.01 (-0.03, 0.01)	-
Sodium	-0.01 (-0.03, 0.01)	-0.01 (-0.03, 0.01)	-
Add sugar	0.03 (0.01, 0.05) *	0.03 (0.01, 0.05) *	-

Model adjusted for age, sex, race, education, income, total energy intake, alcohol intake, smoking, physical activity, BMI, self-reported hypertension, self-reported diabetes, and self-reported cardiovascular disease
*p<0.05
